# Circular RNA circGRAMD1B inhibits gastric cancer progression by sponging miR-130a-3p and regulating PTEN and p21 expression

**DOI:** 10.18632/aging.102414

**Published:** 2019-11-13

**Authors:** Xinglong Dai, Xiong Guo, Jianjun Liu, Anqi Cheng, Xudong Peng, Lang Zha, Ziwei Wang

**Affiliations:** 1 Department of Gastrointestinal Surgery, Laboratory Research Center, The First Affiliated Hospital of Chongqing Medical University, Chongqing 400010, PR China

**Keywords:** gastric cancer, circGRAMD1B, miR-130a-3p, PTEN, p21

## Abstract

Circular RNAs (circRNAs) have emerged as essential regulators and biomarkers of various cancers. However, the effects of a novel circRNA termed circGRAMD1B in human gastric cancer (GC) remain unclear. A microarray was used to screen circRNA expression in GC. Quantitative real-time PCR was used to detect the expression of circGRAMD1B. Gain- and loss- of-function experiments were performed to investigate the biological functions of circGRAMD1B in vitro and vivo. Bioinformatics analysis, fluorescence in situ hybridization, dual-luciferase reporter assay, RNA immunoprecipitation, RNA pull-down assay, and rescue experiments were conducted to confirm the underlying mechanisms of competitive endogenous RNAs (ceRNAs). We screened differentially expressed circRNAs and found that circGRAMD1B expression was downregulated in GC tissues and cell lines. Functionally, circGRAMD1B acted as an anti-oncogene and inhibited the proliferation, migration, and invasion abilities of GC cells. Then, we verified that circGRAMD1B served as a sponge that targeted miR-130a-3p in GC cells; circGRAMD1B alleviated GC cell proliferation, migration, and invasion by targeting miR-130a-3p. A mechanistic analysis showed that PTEN and p21 were involved in circGRAMD1B/miR-130a-3p axis-inhibited GC tumorigenesis. Our findings suggest that circGRAMD1B plays an important role in GC progression by regulating miR-130a-3p-PTEN/p21, which may provide a potential biomarker and therapeutic target for GC.

## INTRODUCTION

Gastric cancer (GC) is one of the most common malignant tumors and the third most frequent cause of cancer-related death worldwide [1]. Despite current advances in surgical techniques, radiation, and chemotherapy strategies, the therapeutic effectiveness of advanced GC has not shown obvious improvement, and the 5-year survival rate is still dismal. The complexity and pathogenic mechanism of GC are believed to be major obstacles; therefore, detailed research into the molecular mechanisms of GC is essential for improving the diagnosis and treatment [2].

Circular RNAs (circRNAs) are a special type of noncoding RNA formed by back-splicing events via exon or intron circularization [3–5]. Emerging evidence has shown that circRNAs act as miRNA sponges to modulate gene transcription and interact with RNA-binding proteins (RBPs) involved in tumorigenesis [6, 7]. Studies have also confirmed that circRNAs participate in various biological and pathological processes, such as proliferation, migration, and invasion [8, 9]. These reports suggest that circRNAs gradually provide a potential perspective on cancer diagnosis and treatment. However, the specific function and molecular mechanism of most circRNAs in human GC remain mostly unknown. In this study, we aimed to identify circRNAs that may be involved in the pathology of GC using circRNA microarrays (Capitalbio, China). We screened and determined the expression and functions of circGRAMD1B (circBase ID: hsa_circ_0004798) derived from GRAM domain-containing 1B (GRAMD1B) in GC and examined the detailed mechanism of this circRNA in GC progression.

Multiple studies have claimed that lncRNAs, circRNAs, and pseudogenes can serve as miRNA “sponges” by sharing common miRNA response elements (MREs) to regulate gene expression [10, 11]. Presently, the competing endogenous RNA (ceRNA) regulation model has become an essential mechanism in various cancers [12, 13]. In this study, we designed a series of functional and molecular assays to explore the ceRNA mechanism of circGRAMD1B and found that phosphatase and tensin homolog (PTEN) and cyclin dependent kinase inhibitor 1A (CDKN1A (p21, Cip1)) constitute a ceRNA regulatory network for the circGRAMD1B/miR-130a-3p axis in GC.

## RESULTS

### Identification and characterization of circGRAMD1B in GC via a microarray analysis

**Figure 1:**
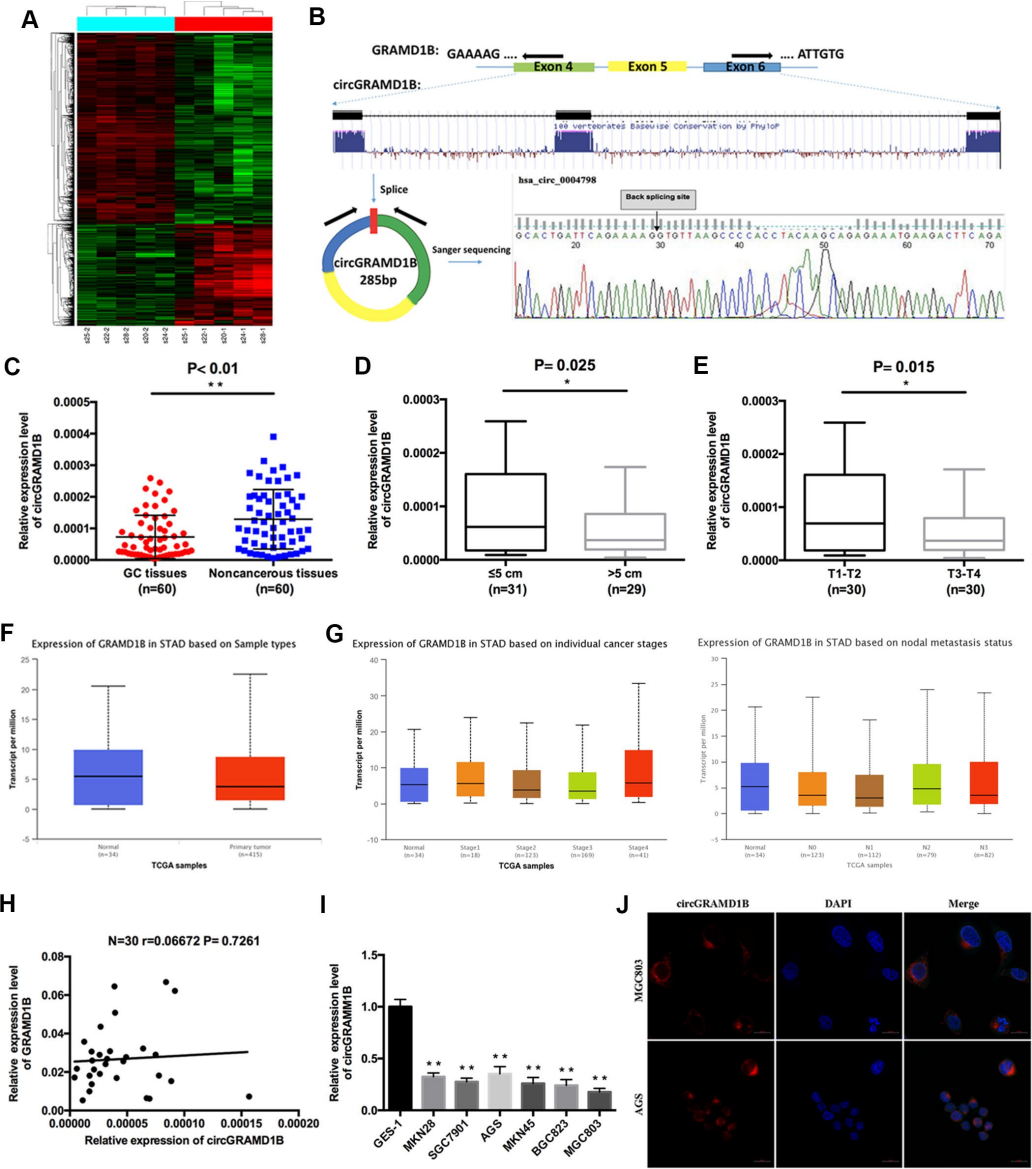
**Characterization of circGRAMD1B in human GC tissues and cell lines.** (**A**) Clustered heat map showing tissue-specific circRNAs, which are displayed on a scale from green (low) to red (high), in five paired human GC tissues and paired noncancerous tissues. (GC tissues: s20-1, s22-1, s24-1, s25-1, s28-1; Paired noncancerous tissues: s20-2, s22-2, s24-2, s25-2, s28-2). (**B**) Schematic representation of circGRAMD1B formation. Arrows represent divergent primers that bind to the genomic region of circGRAMD1B. The splice junction sequence of circGRAMD1B was validated by Sanger sequencing. (**C**) The expression level of circGRAMD1B was detected by qRT-PCR in 60 paired GC tissues and noncancerous tissues. (**D**) qRT-PCR analysis showed that circGRAMD1B expression was lower in > 5cm GC tissues than in ≤ 5cm tissues. (**E**) qRT-PCR analysis showed that circGRAMD1B expression was lower in T3-T4 GC tissues than in T1-T2 tissues. (**F**) TCGA database analysis of GRAMD1B mRNA levels in human GC tissues (n=415) and normal tissues (n=34). (**G**) TCGA database analysis of GRAMD1B mRNA levels in different stages and nodal metastasis status of GC. (**H**) Correlation analysis of circGRAMD1B levels with GRAMD1B mRNA levels in 30 GC tissues. (**I**) Relative expression level of circGRAMD1B in GC cell lines and GES-1 cells using qRT-PCR assay. (**J**) Confocal FISH was performed to determine the cellular location of circGRAMD1B in AGS and MGC803 cells. (Values are shown as the mean ± standard error of the mean based on three independent experiments. *P < 0.05, **P < 0.01).

A total of 5508 differentially expressed circRNA candidates were identified in the circRNA microarray, including 1914 (34.74%) upregulated and 3594 (65.25%) downregulated circRNAs in GC. A clustered heat map in Figure 1A shows the differentially expressed circRNAs. PCR analysis of the differentially expressed circRNAs in a small amount of paired GC tissues and noncancerous tissues indicated that hsa_circ_0004798 (circGRAMD1B) was one of the greatest differentially expressed circRNAs. We then explored circGRAMD1B formation and found that circGRAMD1B, located at the chromosome chr11:123464789-123466745, with a molecular weight of 285 bp, was formed from exons 4, 5 and 6 of GRAMD1B using a bioinformatics method. Sanger sequencing of the PCR products also confirmed the presence of a splice junction in circGRAMD1B (Figure 1B). The qRT-PCR assay indicated that the expression level of circGRAMD1B was significantly decreased in 60 paired GC tissues compared with that in paired noncancerous tissues (Figure 1C). The median expression level was taken as a cut-off value, and the 60 GC patients were divided into two groups, the low circGRAMD1B expression group and the high circGRAMD1B expression group. The clinicopathological parameters of 60 pairs of patients with GC showed that the expression level of circGRAMD1B was correlated with tumor size (P= 0.025) and T stage (P= 0.015) (Figure 1D, 1E; Supplementary Table 1). Further analyses from the TCGA database showed that GRAMD1B mRNA levels had no statistical difference in 415 GC tissues compared with 34 normal tissues (P= 0.1004; Figure 1F). Meanwhile, GRAMD1B mRNA levels indicated no statistically significant at different stages and nodal metastasis status of GC (Figure 1G) [14]. The correlation analysis showed that GRAMD1B mRNA levels were poorly correlated with circGRAMD1B levels in 30 GC tissues (Figure 1H). circGRAMD1B expression was significantly lower in six GC cell lines than in GES-1 cells (Figure 1I). Besides, FISH assay results revealed that circGRAMD1B was primarily expressed in the cytoplasm (Figure 1J). The above results suggest that the reduced expression of circGRAMD1B is frequently observed in GC and may be involved in GC progression.

### circGRAMD1B is anti-oncogenic and associated with proliferation, migration, and invasion in GC cells

**Figure 2:**
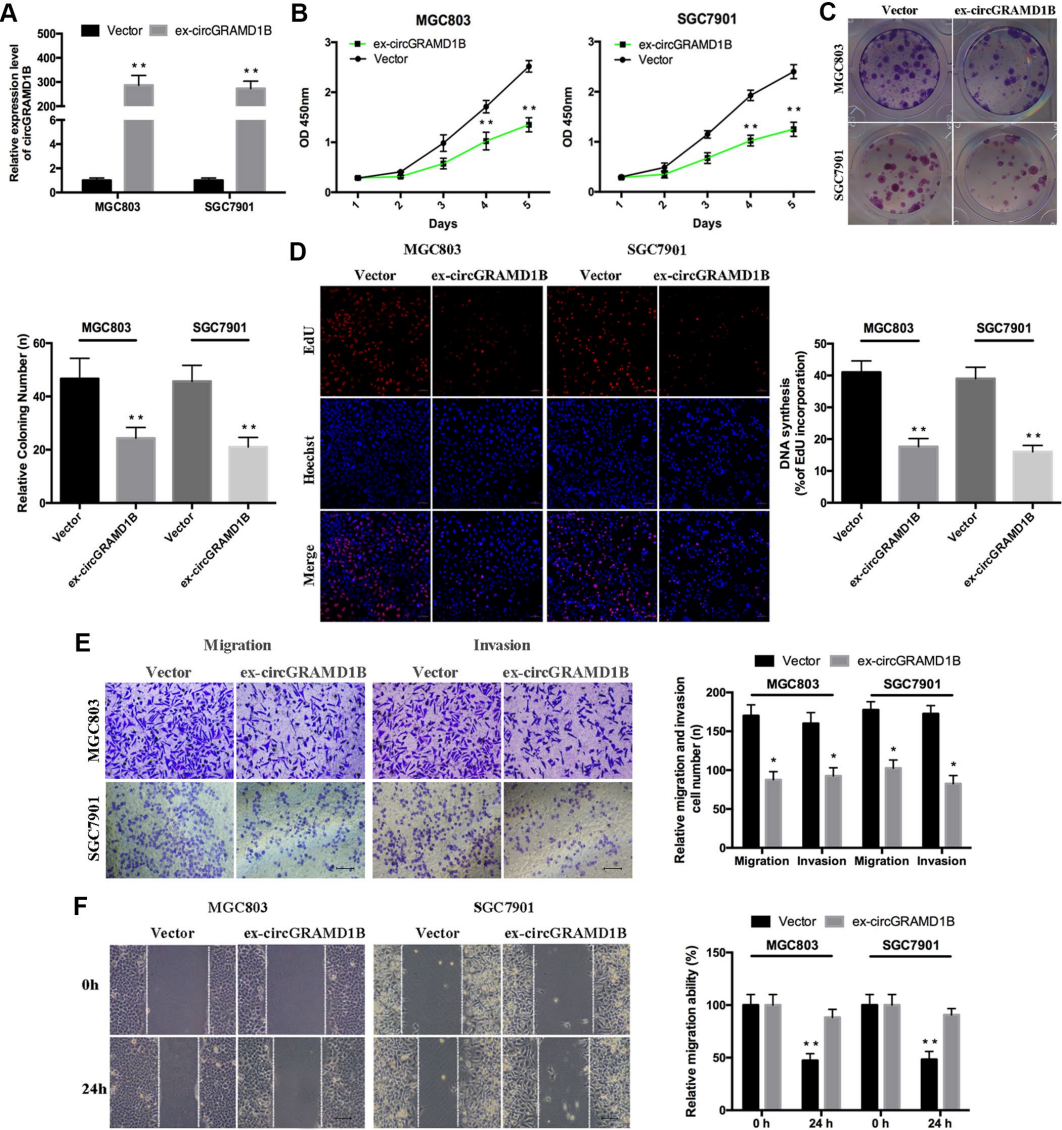
**Overexpression of circGRAMD1B inhibits the proliferation, migration, and invasion of GC cells.** (**A**) qRT-PCR analysis of the transfection efficiency of circGRAMD1B in MGC803 and SGC7901 cells. (**B**–**C**) Cell proliferation abilities were detected by CCK-8 and colony formation assays after the transfection of circGRAMD1B or vector in MGC803 and SGC7901 cells. (**D**) Observation of DNA synthesis in MGC803 and SGC7901 cells transfected with circGRAMD1B overexpressing plasmid or control vector by the EdU assay. (**E**) Cell migration and invasion abilities were measured with transwell assays after the transfection of circGRAMD1B or vector in MGC803 and SGC7901 cells. (**F**) Cell migration ability was determined by a wound-healing assay after the transfection of circGRAMD1B or vector in MGC803 and SGC7901 cells. (Values are shown as the mean ± standard error of the mean based on three independent experiments. *P < 0.05, **P < 0.01).

**Figure 3:**
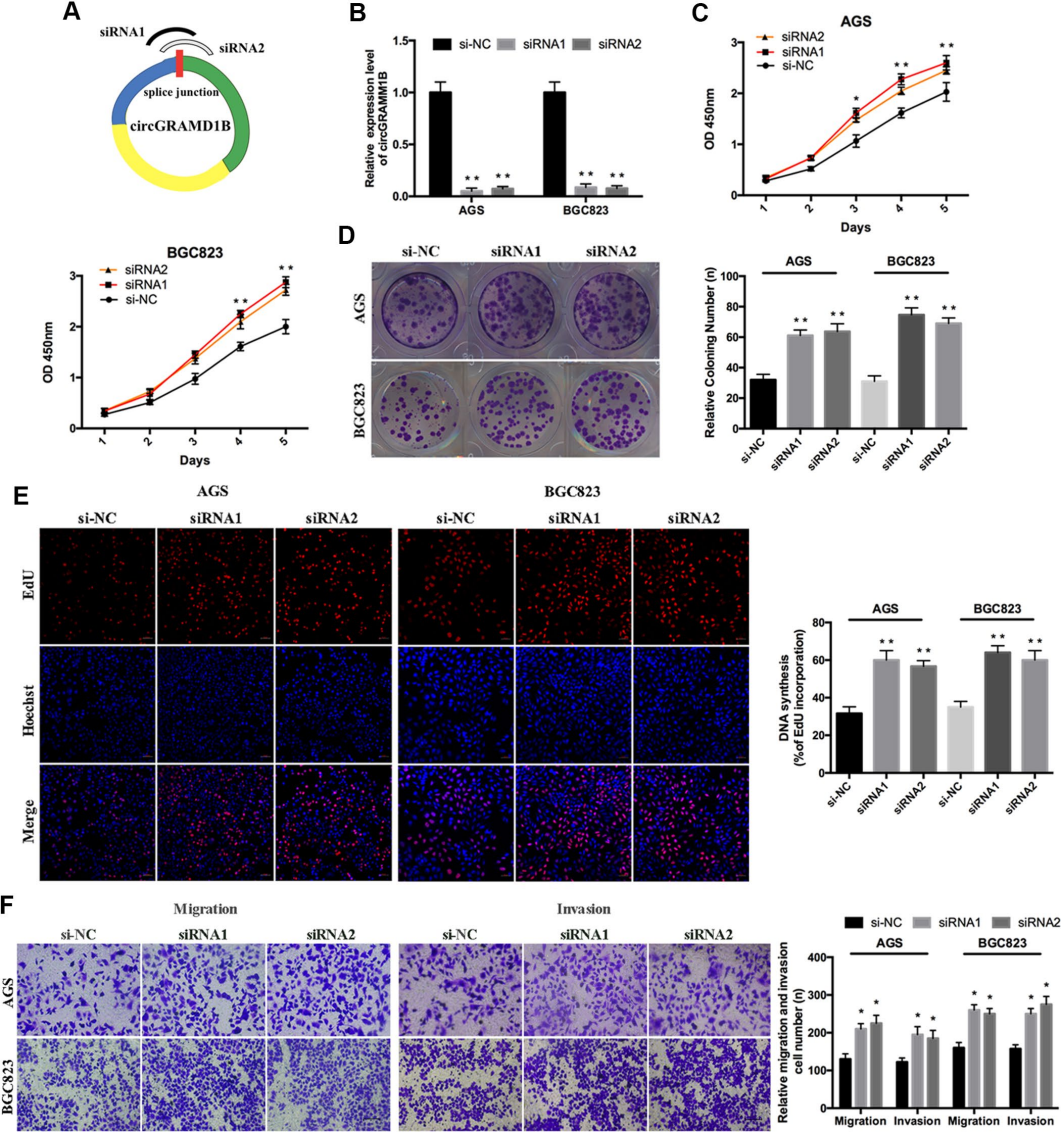
**Silencing of circRAMD1B promotes the proliferation, migration, and invasion of GC cells.** (**A**) Schematic representation of siRNA1/siRNA2 designed to target circGRAMD1B at the back splice junctions. (**B**) qRT-PCR analysis of the silencing efficiency of circGRAMD1B after the transfection for 48 h in AGS and BGC823 cells. (**C**) CCK-8 analysis of the cell viability after the transfection of si-circGRAMD1B or si-NC in AGS and BGC823 cells. (**D**) Colony formation analysis of the cell colony number after the transfection of si-circGRAMD1B or si-NC in AGS and BGC823 cells. (**E**) EdU analysis of the DNA synthesis after the transfection of si-circGRAMD1B or si-NC in AGS and BGC823 cells. (**F**) Transwell analysis of the cell migration and invasive potential after the transfection of si-circGRAMD1B or si-NC in AGS and BGC823 cells. (Values are shown as the mean ± standard error of the mean based on three independent experiments. *P < 0.05, **P < 0.01).

Given that circGRAMD1B is downregulated in GC tissues and cell lines in our study, we further investigated its potential functional role by overexpressing circGRAMD1B in MGC803 and SGC7901 cell lines. MGC803 and SGC7901 cells were transfected with the circGRAMD1B overexpression vector or the control vector, and qRT-PCR was performed to detect the effect of circGRAMD1B overexpression (Figure 2A). The extraction and sequencing of RNA obtained from GC cells overexpressing circGRAMD1B confirmed that the overexpressed circGRAMD1B was circular rather than linear. Functionally, CCK-8, and colony assays demonstrated that overexpression of circGRAMD1B repressed the cell viability and colony formation abilities of MGC803 and SGC7901 cells (Figure 2B, 2C). EdU assay showed that the DNA synthesis of MGC803 and SGC7901 cells was retarded by overexpression of circGRAMD1B, as compared with the control group (Figure 2D). Transwell migration and invasion assays showed that the overexpression of circGRAMD1B significantly suppressed the migration and invasion abilities of MGC803 and SGC7901 cells (Figure 2E). The wound-healing assay also indicated that the cell migration capabilities of MGC803 and SGC7901 cells were significantly weakened by overexpression of circGRAMD1B (Figure 2F). Besides, we further investigated the potential functional role of circGRAMD1B through loss-of-function experiments in GC cell lines. We analyzed the siRNA1/siRNA2 sequence of circGRAMD1B at splice junctions accordingly (Figure 3A). AGS and BGC823 cells were transfected with circGRAMD1B siRNA1/siRNA2 or the si-NC for 48 h, and then qRT-PCR was performed to determine the silencing effect of circGRAMD1B (Figure 3B). Functionally, CCK-8, colony formation, and EdU assays indicated that cell proliferation ability and colony formation were significantly increased after the silencing of circGRAMD1B (Figure 3C–3E). The silencing of circGRAMD1B significantly promoted the migration and invasion abilities of AGS and BGC823 cells (Figure 3F). These results of the in vitro experiments strongly suggest that circGRAMD1B is a novel tumor suppressor in GC progression.

### Prediction of miRNAs and mRNAs targeted by circGRAMD1B

**Figure 4:**
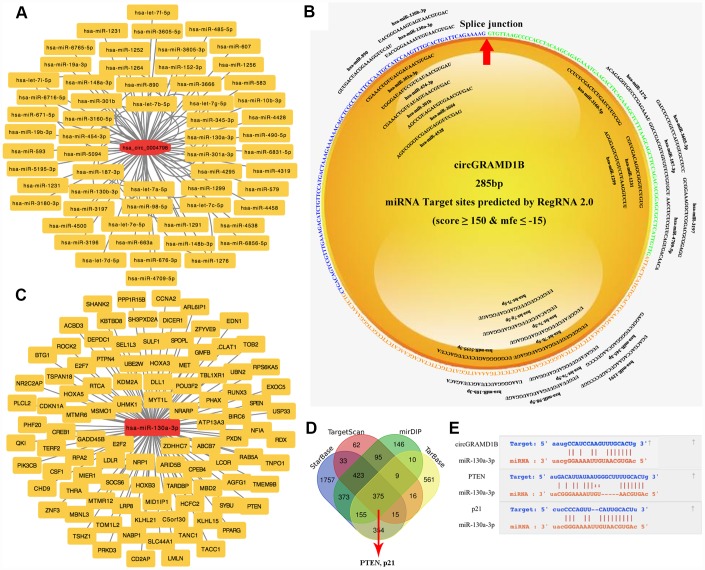
**Prediction of circRNA-miRNA-mRNA associations.** (**A**) A magnified network comprising circGRAMD1B and its target miRNAs is presented. (**B**) The binding sites of miRNAs and circGRAMD1B were predicted by RegRNA 2.0. (**C**) The panorama network consists of the target genes and miR-130a-3p. Only 100 reliable target genes were added to our predicted picture. (**D**) Venn diagram showing the 375 commonly expressed mRNA targets of miR-130a-3p obtained from four publicly available profile databases (DIANA TarBase, TargetScan, mirDIP, and StarBase database). Two mRNA targets of miR-130a-3p were identified: PTEN and p21. (**E**) Schematic representation of the potential binding sites of circGRAMD1B with miR-130a-3p and miR-130a-3p with PTEN/p21.

Previous studies have reported that cytoplasmic circRNAs can function as miRNA sponges and then regulate gene expression [8, 10]. In this study, by bioinformatics analysis and predictive websites, we detected 59 miRNAs that share the MREs of circGRAMD1B (Figure 4A), which might bind. As shown in Figure 4B (RegRNA 2.0 prediction, miRNA Target Sites: score ≥ 150 and mfe ≤ -15), we found that miR-130a-3p had a putative binding site with circGRAMD1B. A miRNA microarray of human GC from the TCGA database (DIANA: mirExTra 2.0) was used to analyze the miRNA expression profile in paired GC tissues and noncancerous tissues. Based on the fold change and P-value, we found that hsa-miR-130a-3p, hsa-miR-130b-3p, and hsa-miR-98-5p were the three miRNAs with the most significant differential expression. miR-130a-3p showed a higher probability of binding to circGRAMD1B on most predictive websites; therefore, we selected miR-130a-3p as a sponge for circGRAMD1B for further study. Furthermore, according to the ceRNA regulatory model in tumor cells, the DIANA TarBase, TargetScan, mirDIP, and StarBase database showed supported interactions between the experimental mRNAs and hsa-miR-130a-3p. Although miR-130a-3p was predicted to have approximately 375 downstream target genes, only 100 reliable target genes, including PTEN and p21, were added to our predicted picture (Figure 4C, 4D). In total, we performed integrated bioinformatics analysis and found that circGRAMD1B and the 3′-UTR of both PTEN/p21 share the same MREs of miR-130a-3p, which suggested an association between circGRAMD1B and PTEN/p21 in GC (Figure 4E).

### circGRAMD1B acts as a sponge and targets miR-130a-3p in GC cells

**Figure 5:**
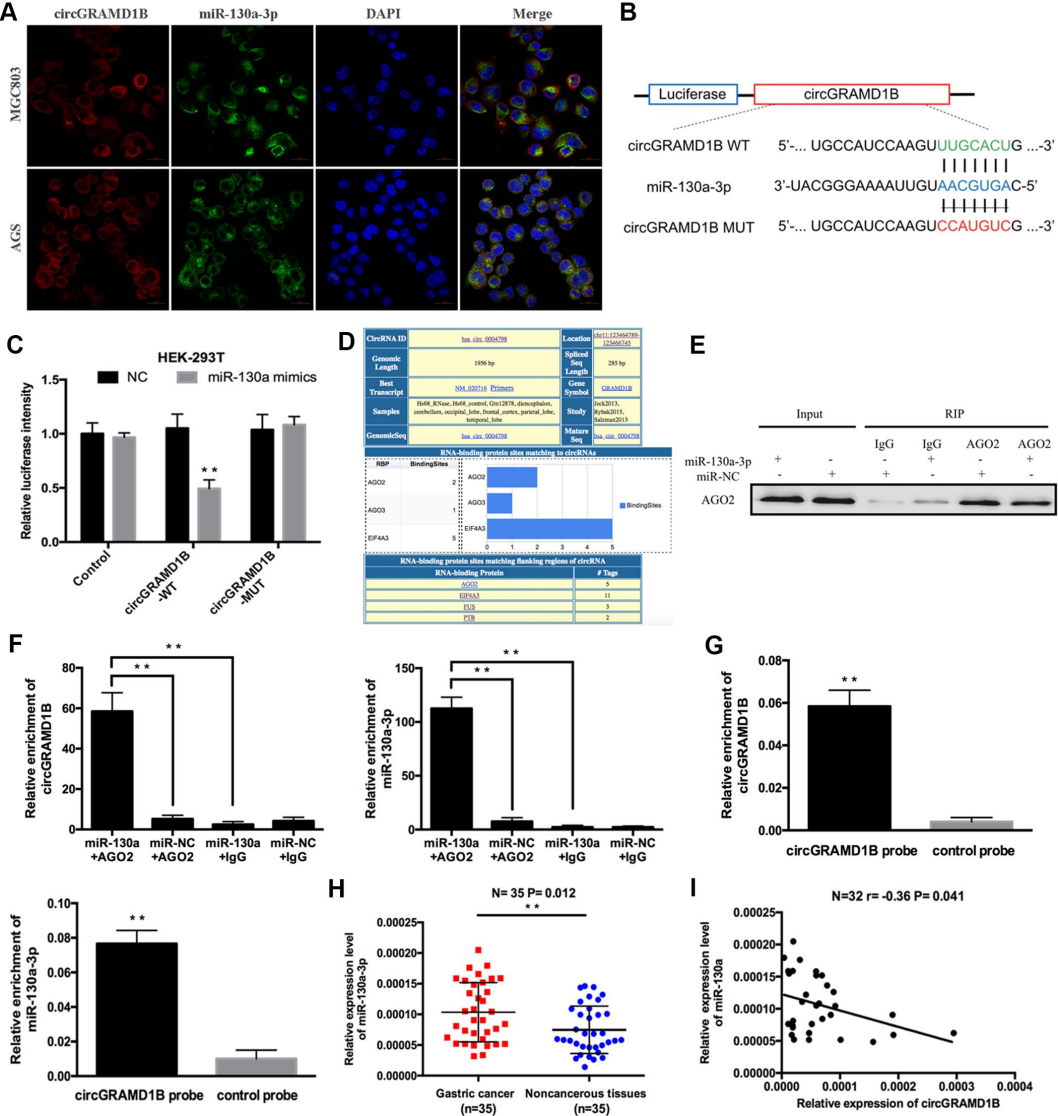
**circGRAMD1B acts as a molecular sponge for miR-130a-3p in GC cells.** (**A**) Colocalization of circGRAMD1B and miR-130a-3p was measured using FISH in MGC803 and AGS cells. (**B**) Schematic illustration of circGRAMD1B-WT and circGRAMD1B-MUT luciferase reporter vectors. (**C**) A luciferase reporter assay was performed to detect the activities of circGRAMD1B -WT and -MUT in HEK-293T cells cotransfected with miR-130a-3p mimics or the miR-NC. (**D**) Screenshot of “Circular RNA Interactome” for has_circ_0004798 (circGRAMD1B), showing RBPs binding in different regions of this circRNA. (**E**–**F**) Anti-AGO2 RIP was performed in AGS cells after transfection with the miR-130a-3p mimics or miR-NC, followed by western blot and qRT-PCR analyses to detect AGO2 protein, circGRAMD1B, and miR-130a-3p. (**G**) RNA pull-down assays were performed in AGS cells, followed by qRT-PCR to detect the enrichment of circGRAMD1B and miR-130a-3p. (**H**) Relative expression level of miR-130a-3p was upregulated in GC tissues compared with paired noncancerous tissues according to qRT-PCR analysis. (**I**) Pearson correlation analysis of circGRAMD1B and miR-130a-3p expression in 32 GC tissues. (Values are shown as the mean ± standard error of the mean based on three independent experiments. *P < 0.05, **P < 0.01).

To identify the downstream miRNAs of circGRAMD1B, a FISH assay was performed, which suggested that circGRAMD1B and miR-130a-3p were colocalized in the cytoplasm of MGC803 and AGS cells (Figure 5A). A luciferase reporter assay was performed to confirm the interaction between circGRAMD1B and miR-130a-3p (Figure 5B). The relative luciferase activity of circGRAMD1B-WT was obviously reduced after cotransfection of the miR-130a-3p mimics but did not change the activity of circGRAMD1B-MUT, which suggests that miR-130a-3p is a direct target of circGRAMD1B (Figure 5C). Additionally, according to previous studies [15, 16], there is a prevalent phenomenon that miRNAs generally inhibit translation or degrade mRNA by binding with Argonaute 2 (AGO2) protein. Bioinformatics analysis indicated that circGRAMD1B has a binding site for AGO2 protein; therefore, we further performed AGO2-RIP assay in GC cell lines (Figure 5D). An anti-AGO2 RIP assay indicated that AGO2, circGRAMD1B, and miR-130a-3p were all efficiently pulled down by anti-AGO2 antibodies compared with that by IgG (Figure 5E), and both circGRAMD1B and miR-130a-3p were significantly enriched in cells transfected with miR-130a-3p mimics compared with that of the miR-NC group (Figure 5F). We further performed an RNA pull-down assay with specific biotin-labeled circGRAMD1B probes. Interestingly, specific enrichment of circGRAMD1B and miR-130a-3p was detected by qRT-PCR in the circGRAMD1B probe group compared with that in the control probe group (Figure 5G). Subsequently, qRT-PCR assay evaluated the expression level of miR-130a-3p in GC tissues, and the results showed that miR-130a-3p was upregulated in 35 paired GC tissues compared with that in paired noncancerous tissues (Figure 5H). In addition, there was also a negative correlation between circGRAMD1B and miR-130a-3p in 32 GC tissues (r= -0.36, P= 0.041, Figure 5I).

### circGRAMD1B alleviates GC cell proliferation, migration, and invasion by targeting miR-130a-3p

**Figure 6:**
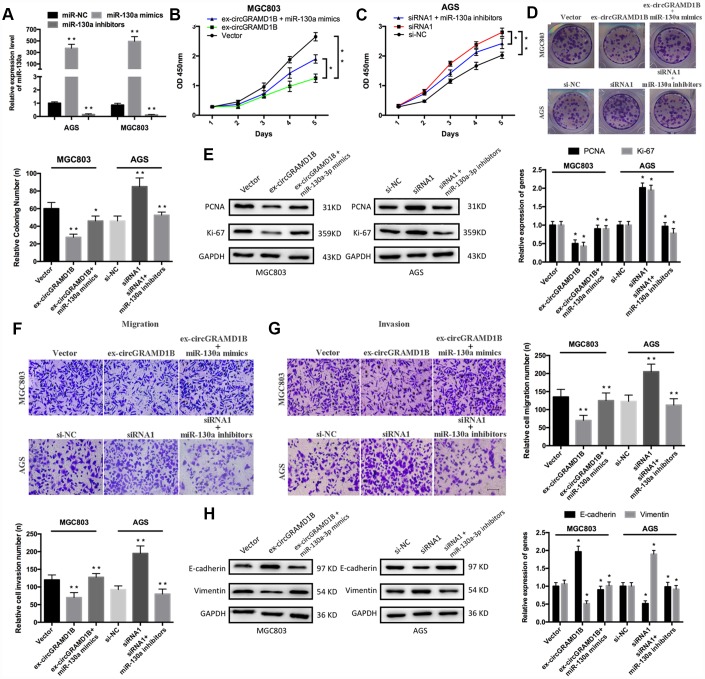
**circGRAMD1B alleviates GC cell proliferation, migration, and invasion by targeting miR-130a-3p.** (**A**) miR-130a-3p overexpression or silencing was performed in MGC803 and AGS cells with miR-130a-3p mimics or inhibitors, respectively. (**B**–**C**) CCK-8 assays were used to assess the proliferation abilities of the transfected MGC803 and AGS cells. MGC803 cells were transfected with the miR-NC, the circGRAMD1B-overexpressing plasmid, or the circGRAMD1B-overexpressing plasmid and miR-130a-3p mimics; AGS cells were transfected with the miR-NC, circGRAMD1B siRNA1^#^ or circGRAMD1B siRNA1^#^ and miR-130a-3p inhibitors. (**D**) Colony formation assays were used to evaluate the proliferation abilities of the transfected MGC803 and AGS cells. (**E**) Western blot assays were used to analyze the protein expression levels of PCNA and Ki-67 in transfected MGC803 and AGS cells. (**F**–**G**) Transwell assays were performed to evaluate the migration and invasion abilities of transfected MGC803 and AGS cells. (**H**) Western blot assays were used to analyze the protein expression levels of E-cadherin and Vimentin in transfected MGC803 and AGS cells. (Values are shown as the mean ± standard error of the mean based on three independent experiments. *P < 0.05, **P < 0.01).

To further investigate the roles of miR-130a-3p and circGRAMD1B in GC progression, we performed rescue experiments, such as CCK-8, colony formation, and Transwell assays, to evaluate the effects of the circGRAMD1B/miR-130a-3p axis in GC cells. miR-130a-3p expression was increased or decreased after MGC803 and AGS cells were transfected with miR-130 mimics or inhibitors, respectively (Figure 6A). CCK-8 assay indicated that circGRAMD1B inhibited MGC803 cell proliferation and that miR-130a-3p mimics attenuated this inhibition (Figure 6B). Moreover, the silencing of circGRAMD1B promoted AGS cell proliferation, and the miR-130a-3p inhibitors weakened this promotion (Figure 6C). The cloning formation assay showed similar proliferation effects (Figure 6D). circGRAMD1B also decreased the expression levels of proliferation-associated markers (PCNA and Ki-67), and miR-130a-3p mimics reversed this tendency. The silencing of circGRAMD1B increased the expression levels of proliferation-associated markers (PCNA and Ki-67), and miR-130a-3p inhibitors reversed this tendency (Figure 6E). Furthermore, Transwell analysis showed that circGRAMD1B reduced MGC803 cell migration and invasion and that miR-130a-3p mimics attenuated this suppression. Moreover, the silencing of circGRAMD1B increased AGS cell migration and invasion, and miR-130a-3p inhibitors weakened this acceleration (Figure 6F, 6G). Western blot assays revealed that the circGRAMD1B/miR-130a-3p axis regulated the expression of epithelial-mesenchymal transition (EMT) markers (E-cadherin and Vimentin) in GC cells (Figure 6H). These data indicated that circGRAMD1B functions as a ceRNA to suppress GC progression partly by abolishing the oncogenic effect of miR-130a-3p.

### circGRAMD1B regulates the progression of GC by modulating miR-130a-3p/PTEN/p21 axis

**Figure 7:**
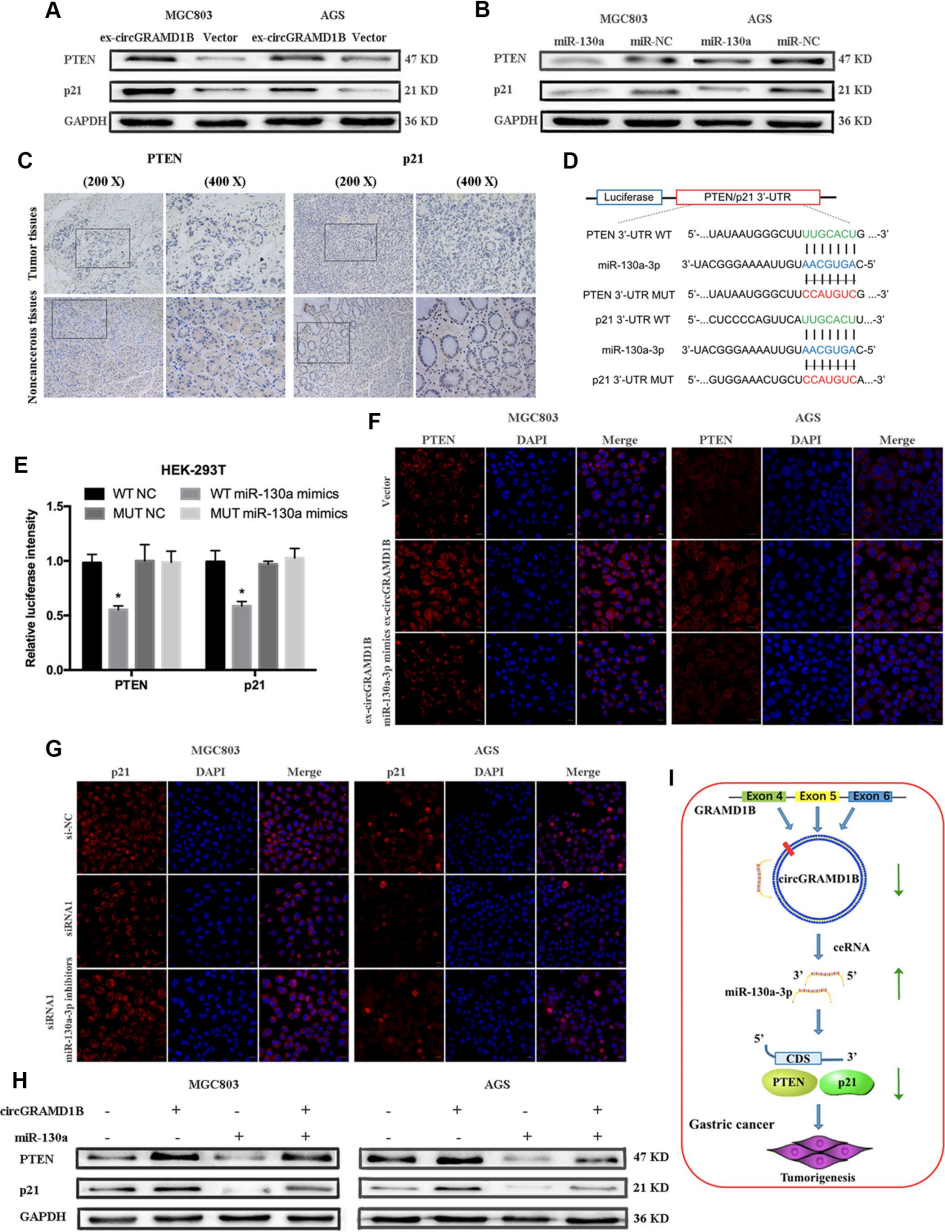
**circGRAMD1B inhibits the proliferation and invasion of GC cells by modulating miR-130a-3p/PTEN/p21 axis.** (**A**) Western blot assays showed that circGRAMD1B upregulated the protein expression levels of PTEN and p21 in transfected MGC803 and AGS cells. (**B**) Western blot assays showed that miR-130a-3p decreased the protein expression levels of PTEN and p21 in transfected MGC803 and AGS cells. (**C**) PTEN and p21 expression was detected via IHC in GC tissues and paired noncancerous tissues. (**D**) Schematic illustration of PTEN/p21 -3′ UTR-WT and -3′ UTR-MUT luciferase reporter vectors. (**E**) Luciferase reporter assays demonstrated that PTEN and p21 are direct targets of miR-130a-3p mimics. (**F**–**G**) Relative protein expression level of PTEN and p21 were assessed by IF after GC cells transfected with circGRAMD1B-overexpressing plasmid or the silencing siRNA1, and miR-130a-3p mimics or inhibitors. (**H**) Western blot assays showed that miR-130a-3p could partly decrease the protein expression levels of PTEN and p21, which were promoted by circGRAMD1B. (**I**) Schematic diagram of the regulatory mechanism of circGRAMD1B/ miR-130a-3p/ PTEN/p21 axis in the inhibition of GC progression. (Values are shown as the mean ± standard error of the mean based on three independent experiments. *P < 0.05, **P < 0.01).

We further explored which downstream molecule might be affected by the circGRAMD1B/miR-130a-3p axis to regulate the GC phenotype. Based on results from the combination of several predictive websites, we found that PTEN and p21 were the target genes of miR-130a-3p and may be regulated by circGRAMD1B in GC. We selected PTEN and p21 as the possible downstream target genes of miR-130a-3p. First, the results showed that PTEN and p21 expression had the same tendency as that of circGRAMD1B (Figure 7A), while miR-130a-3p expression reduced the expression of PTEN and p21 (Figure 7B). These results suggest that PTEN and p21 may partly serve as downstream factors in the circGRAMD1B/miR-130a-3p axis. Then, we confirmed the expression of PTEN and p21 in four paired GC tissues and paired noncancerous tissues via IHC, and the results indicated that they exhibited low expression in GC tissues (Figure 7C). Consequently, a luciferase reporter assay was performed, and cotransfection of miR-130a-3p mimics and wild reporter plasmids strongly reduced luciferase activity. Conversely, cotransfection of miR-130a-3p mimics and mutated vectors showed no obvious effect on luciferase activity (Figure 7D, 7E). Indeed, IF (Figure 7F, 7G) and Western blot (Figure 7H) assays revealed that circGRAMD1B significantly regulates the expression of PTEN and p21, respectively, via miR-130a-3p. These data provide evidence that the role of circGRAMD1B in suppressing GC progression is primarily dependent on the miR-130a-PTEN/p21 axis (Figure 7I).

### The effects of circGRAMD1B on in vivo tumor growth and lung metastasis

**Figure 8:**
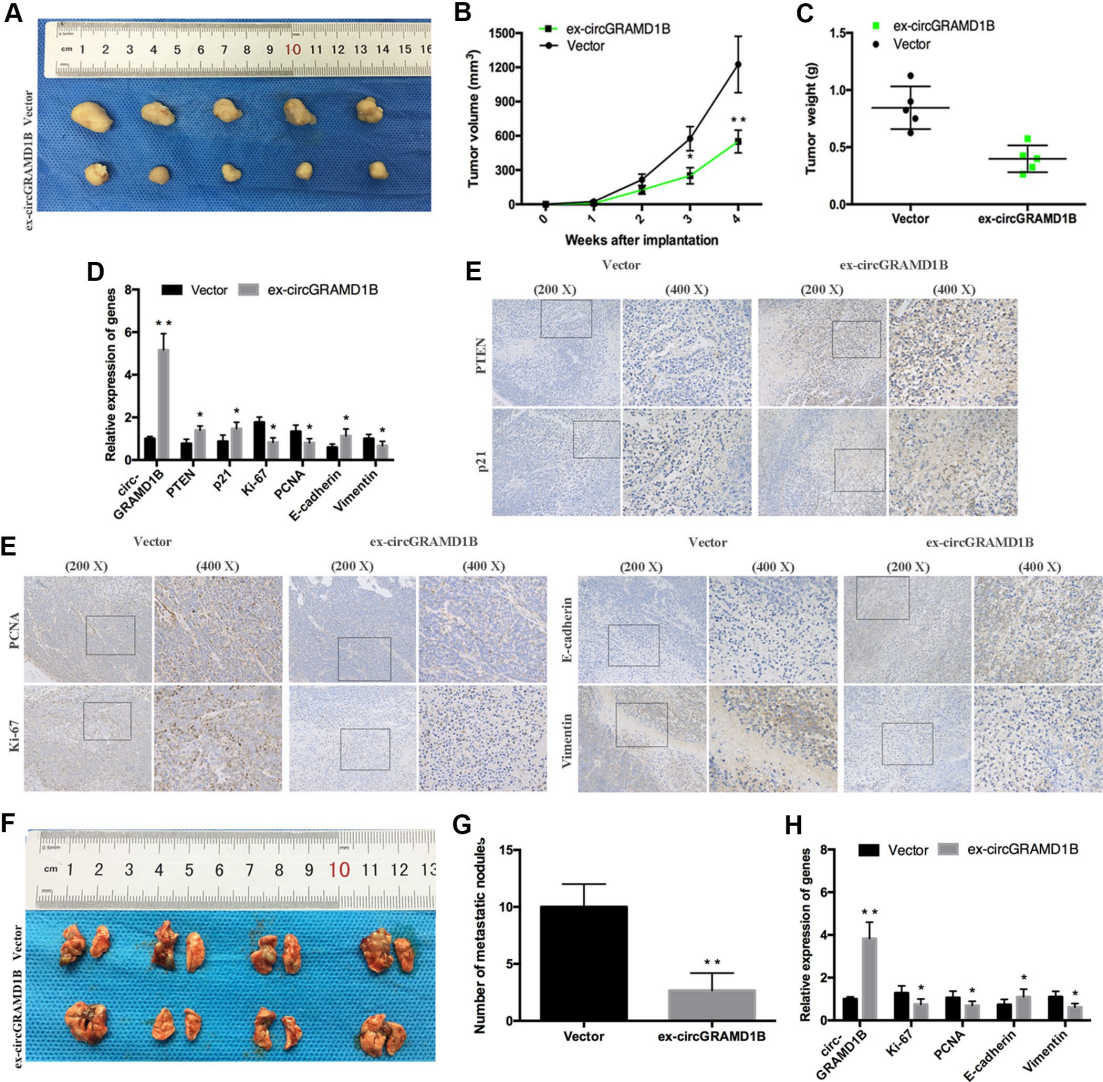
**circGRAMD1B inhibits GC growth and metastasis in vivo.** (**A**) Representative images of xenograft tumors induced by circGRAMD1B overexpressing vector and control vector-transfected MGC803 cells (five mice per group). (**B**) A growth curve of the tumor growth in circGRAMD1B overexpressing vector and control vector-transfected MGC803 cells. (**C**) Tumor weights of the xenograft tumors in circGRAMD1B overexpressing vector and control vector-transfected MGC803 cells. (**D**) Expressions levels of circGRAMD1B, PTEN, p21, Ki-67, PCNA, E-cadherin, and Vimentin in xenograft tumors were determined by qRT-PCR assays. (**E**) Expression levels of PTEN, p21, Ki-67, PCNA, E-cadherin, and Vimentin were shown by IHC staining in representative xenograft tumors. (**F**) Representative images of lung metastatic nodules in the circGRAMD1B overexpression group and control group are indicated by white arrows. (**G**) The number of tumor nodules on lung surfaces from the circGRAMD1B overexpression group and control group. (**H**) qRT-PCR was used to detect the expression levels of circGRAMD1B, Ki-67, PCNA, E-cadherin, and Vimentin in lung metastasis from nude mice in the circGRAMD1B overexpressing group and control group. (Values are shown as the mean ± standard error of the mean based on three independent experiments. *P < 0.05, **P < 0.01).

To determine the effects of circGRAMD1B on tumor growth and lung metastasis in vivo, MGC803 cells transfected with stable circGRAMD1B vector or control vector were injected subcutaneously into nude mice. Consistent with the in vitro data, a smaller volume and lighter weight of xenograft tumors were observed in the circGRAMD1B overexpressing group compared to the control group (Figure 8A–8C). Additionally, overexpression of circGRAMD1B triggered a reduction of Ki-67, PCNA, and Vimentin expression, while an increase of circGRAMD1B, PTEN, p21, and E-cadherin mRNA level in excised tumor masses (Figure 8D). The IHC assay showed that in the dissected tumors, circGRAMD1B overexpression enhanced the immunostaining of PTEN, p21, and E-cadherin expression and reduced the immunostaining of Ki-67, PCNA, and Vimentin expression (Figure 8E). We then intravenously injected the abovementioned cells into nude mice to construct a lung metastasis model. As expected, the number of metastatic lesions in the lungs was lower in the circGRAMD1B overexpression group than in the control group (Figure 8F, 8G). In the lung metastasis model, the mRNA levels of Ki-67, PCNA, and Vimentin were decreased in the circGRAMD1B overexpression group compared with the control group, while E-cadherin level was increased in the circGRAMD1B overexpression group (Figure 8H). The results of the in vivo experiments also suggest that circGRAMD1B is a novel tumor suppressor in GC progression and metastasis.

## DISCUSSION

Previous studies have demonstrated that GC is challenging for the surgeon due to its rapid proliferation and infiltrative growth, leading to invasion into adjacent tissue and resulting in incomplete resection and recurrence. Therefore, it is crucial to study the high proliferation and invasion of GC. circRNAs are promising potential biomarkers for the disease because of their unique structure, high stability, and specific expression patterns [17]. Recently, circRNAs are dysregulated in multiple cancers, including GC. For example, the circular RNA PRMT5 promotes the metastasis of bladder carcinoma by sponging miR-30c and induces epithelial-mesenchymal transition [18]. The circular RNA UBXN7 inhibits cell growth and invasion in BC by sponging miR-1247-3p and regulating B4GALT3 expression [19]. Therefore, we performed circRNA microarray screening and selected a new target to verify its specific function and mechanism. As a result, we found a novel circRNA, circGRAMD1B, that has never been reported in cancer. Next, we demonstrated that circGRAMD1B was significantly decreased in GC tissues and cell lines and that lower circGRAMD1B expression in GC tissues was correlated with tumor size and T stage. Additionally, both circGRAMD1B and GRAMD1B mRNA are derived from the same precursor RNA of GRAMD1B gene through back splicing and canonical linear splicing, respectively. We observed that circGRAMD1B levels in GC tissues were poorly correlated with the levels of GRAMD1B mRNA. This discrepancy is complicated and might be caused by tumor heterogeneity or alternative splicing events in circRNAs [20]. The functional experiment showed that circGRAMD1B inhibited tumor proliferation, metastasis, and invasion both in vitro and in vivo, suggesting that circGRAMD1B can act as a tumor suppressor in GC. These findings indicate that circGRAMD1B acts as an anti-oncogene in the progression of GC, and it has the potential to become a novel biomarker and therapeutic target for GC patients.

Growing evidence indicated that circRNAs could serve as sponges for miRNAs to regulate the expression of miRNA target genes in multiple human cancers [21, 22]. In our study, we demonstrated that circGRAMD1B contained the MRE of miR-130a-3p through bioinformatics analyses. FISH assay displayed that circGRAMD1B and miR-130a-3p were co-located in the cytoplasm of GC cells. Therefore, we inferred that circGRAMD1B might play an anti-oncogenic role via sponging miR-130a-3p in GC. Although miR-130a-3p is a crucial regulator of human cancer progression [23], few studies on the function and mechanism of both miR-130a-3p and circRNAs have been conducted to date. In terms of the mechanism involved, dual-luciferase reporter, anti-AGO2 RNA immunoprecipitation and RNA pull-down assays confirmed that circGRAMD1B could interact with miR-130a-3p directly. Subsequently, our study provided evidence that miR-130a-3p was significantly upregulated in GC tissues and was inversely correlated with circGRAMD1B expression in GC. Our results demonstrated that circGRAMD1B as an anti-oncogene by sponging miR-130a-3p in GC and revealed the significance of the interaction between circGRAMD1B and miR-130a-3p in GC progression.

Many studies have reported that circRNAs mainly act as miRNA sponges and result in a loss of miRNA function accompanied by increased levels of their endogenous targets [24, 25]. In our study, bioinformatics analysis indicated that miR-130a-3p contains a potential binding site for PTEN/p21, suggesting that circGRAMD1B may specifically sponge miR-130a-3p binding to the PTEN/p21 gene in GC. Then, a dual-luciferase reporter assay confirmed that miR-130a-3p could directly target the 3′-UTR of PTEN/p21. circGRAMD1B increased the expression of PTEN/p21, as we hypothesized, suggesting a regulatory network between circGRAMD1B and PTEN/p21. IF assays and Western blot assays revealed that circGRAMD1B regulates the expression of PTEN and p21 via miR-130a-3p in GC cells. A series of experiments presented a new regulatory axis formed by circGRAMD1B-miR-130a-PTEN/p21 in GC. However, there are limitations in our current study. Our future work will focus on the following explorations: Validate the expression of circGRAMD1B in a larger cohort of GC patients. More GC patients need to be followed up for retrospective analysis, aimed at finding out the relationship between circGRAMD1B and the clinical outcomes and prognosis of GC patients.

Our study can be summarized by the following major findings: 1. We identified a novel circular RNA, circGRAMD1B, that acts as an anti-oncogene and inhibits GC proliferation, migration, and invasion in vitro and in vivo. 2. We found that circGRAMD1B was generated from exons 4, 5, and 6 of GRAMD1B mRNA. 3. We suggest that circGRAMD1B can directly bind to miR-130a-3p and regulate its expression. 4. We demonstrated that PTEN and p21 are target genes of the circGRAMD1B/miR-130a-3p axis in GC cells. Therefore, we suggested a novel circGRAMD1B/miR-130a-3p/PTEN, p21 regulatory network in GC, which may provide a potential biomarker and therapeutic target for the management of GC.

## MATERIALS AND METHODS

### Clinical samples

A total of 60 paired GC tissues and noncancerous tissues from patients with GC who had surgically undergone radical surgery were collected at the First Affiliated Hospital of Chongqing Medical University (Chongqing, China) between December 2016 and March 2018. The final diagnosis was histopathologically confirmed and staged according to the TNM staging of the American Joint Committee on Cancer (AJCC). The clinical-pathological features of all patients were recorded and are summarized in Supplementary Table 1. Tissues were collected after surgical resection and stored in liquid nitrogen until further use. Additionally, we selected five paired GC tissues to carry out the microarray assay to determine the expression status of circRNAs in GC tissues (CapitalBio Technology Co., Ltd, Beijing, China). The use of tissues for this study was approved by the Clinical Research Ethics Committee of Chongqing Medical University, and all patients agreed to participate in the study and provided written informed consent.

### Cell culture

Human GC cell lines (AGS, SGC7901, MKN28, MKN45, MGC803, and BGC823) were purchased from the Type Culture Collection of the Chinese Academy of Sciences (Beijing, China). An immortalized gastric mucosal epithelial cell line (GES-1) and the human embryonic kidney (HEK)-293T cell line were donated by the Molecular Tumor and Epigenetics Laboratory from the First Affiliated Hospital of Chongqing Medical University (Chongqing, China). Cells were cultured in RPMI-1640 medium (Gibco, Carlsbad, CA, USA) or DMEM (Gibco, Carlsbad, CA, USA) supplemented with 10% fetal bovine serum (FBS; Biological Industries, Beit-Haemek, Israel). All cell lines were maintained in a humidified cell incubator at 37 °C with an atmosphere of 5% CO_2_.

### RNA extraction and quantitative real-time PCR (qRT-PCR)

Total RNA was isolated from GC tissues, and cells were extracted using TRIzol reagent (Takara, Dalian, China) and then reverse transcribed into cDNA by a PrimeScript RT Reagent Kit (Takara) following the manufacturer’s instructions. qRT-PCR was conducted on a Bio-Rad CFX96 system (Bio-Rad, CA, USA) using TB Green Premix Ex Taq (Takara). Divergent primers rather than the more commonly used convergent primers of circGRAMD1B were designed. Primers for GAPDH, circGRAMD1B, GRAMD1B, PTEN, p21, Ki-67, PCNA, E-cadherin, and Vimentin were synthesized by Sangon Biotech (Shanghai, China). Primers for miR-130a-3p and U6 were synthesized by Ribobio (Guangzhou, China). The primer sequences used are shown in Supplementary Table 2. Each sample was run in triplicate, and the data were analyzed using the relative quantification of the 2^-ΔΔCt^ method. The PCR products were further identified through Sanger sequencing by Sangon Biotech (Shanghai, China).

### Vector construction and cell transfection

To overexpress circGRAMD1B, a 285 bp fragment of circGRAMD1B was cloned into the pHBLV-CMV-crRNA-EF1-T2A vector, and the plasmid or lentivirus was constructed with the assistance of Hanbio Biotech (Shanghai, China). The procedure for GC cells transfected with a lentivirus was conducted according to the manufacturer’s instructions, and after 48 h post-transfection, cells were selected with puromycin (2 μg/mL) for 2 weeks to construct cell lines with stable circGRAMD1B overexpression. Two individual circGRAMD1B siRNAs (siRNA1# and siRNA2#), a miR-130a-3p mimic, a miR-130a-3p inhibitor, and a scrambled negative control siRNA (si-NC) or miRNA negative control (miR-NC) were purchased from Ribobio (Guangzhou, China). Additionally, GC cells were transfected with the abovementioned oligonucleotides and plasmids using Lipofectamine 2000 (Invitrogen, Carlsbad, CA, USA) according to the manufacturer’s instructions. The nucleotide sequences are listed in Supplementary Table 2. After transfection for 48–72 h, the GC cells were harvested for further experiments.

### Cell proliferation and colony formation assays

For the Cell Counting Kit-8 (CCK-8) assay, the transfected cells were seeded into 96-well plates at a density of 10^3^ cells per well. At 0, 24, 48, 72, and 96 h after seeding, cell proliferation was measured by the CCK-8 (Dojindo Laboratories, Kumamoto, Japan) system according to the manufacturer’s instructions. Briefly, 10 μl of CCK-8 solution was added to each well, and the plate was incubated for 3 h at 37 °C with 5% CO_2_. Each well was evaluated by measuring the absorbance values at a wavelength of 450 nm. For the colony formation assay, 200 transfected cells were seeded into 24-well plates and incubated at 37 °C for 12 days. The colonies were fixed with 4% paraformaldehyde and stained with 0.1% crystal violet, and the visible colonies were imaged and manually counted. For each treatment group, wells were assessed in triplicate, and experiments were independently repeated three times.

### 5-Ethynyl-2′-deoxyuridine (EdU) assay

Proliferating cells were assessed using a 5-ethynyl-2-deoxyuridine (EdU) Labeling/Detection Kit (RiboBio, Guangzhou, China) according to the manufacturer’s protocol. The GC cells were cultured in 96-well plates at a density of 1x10^4^ cells per well and transfected with the circGRAMD1B overexpressing plasmid or the siRNA1/siRNA2 for 48 h. Then, 50 μl/L EdU labeling media were added to the plates, which were incubated for 2 h at 37 °C under 5% CO_2_. After treatment with 4% paraformaldehyde and 0.5% Triton X-100, cells were stained with the anti-EdU working solution. Nuclei were stained with Hoechst 33342 reaction solution. The percentage of EdU-positive cells was calculated from five random fields in three wells under a confocal laser scanning microscopy (LSM800, Carl Zeiss AG, Germany).

### Cell migration/invasion and wound healing assays

For migration assay, the treated GC cells (3 x 10^4^ cells) in 200 μl of serum-free RPMI 1640 medium were seeded in the upper part of each transwell chamber (8 μm pore size, Corning Costar, Cambridge, MA, USA). For the invasion assay, the treated GC cells in 200 μl of serum-free RPMI 1640 medium were seeded in the upper chamber of each insert, which was coated with 50 μl of 2 mg/ml Matrigel (BD Biosciences, NJ, USA). The number of transfected cells passing through the matrigel membrane was normalized to the control membrane (without the coat). The lower chambers were filled with RPMI 1640 medium containing 10% FBS. The cells were incubated at 37 °C with 5% CO_2_ for 48 h for the invasion assay and 24 h for the migration assay. The cells on the lower filter surface were fixed with 4% paraformaldehyde, stained with 0.1% crystal violet, and then photographed. For the wound healing assays, the transfected cells were plated into 6-well plates and cultured until 100% confluence. An artificial scratch was created using a 20 μl pipette tip. At 0 and 24 h after culturing in serum-free medium, wound closure images were captured in the same field. These experiments were performed in triplicate and repeated three times.

### Bioinformatics prediction for circGRAMD1B-miRNA-gene interactions

The circRNA/microRNA/gene interaction was predicted using the following websites: Starbase (http://starbase.sysu.edu.cn/index.php) [26], Circinteractome (https://circinteractome.nia.nih.gov;) [27], Circbank (http://www.circbank.cn;) [28], RegRNA 2.0 (http://regrna2.mbc.nctu.edu.tw;) [29], TargetScan (http://www.targetscan.org/vert_72/) [30], DIANA-TarBase (http://diana.imis.athena-innovation.gr/DianaTools/index.php?r=tarbase/index) [31] and mirDIP (http://ophid.utoronto.ca/mirDIP/) [32]. To establish the circRNA-miRNA-gene network, we searched for MREs on circGRAMD1B and target genes using the bioinformatics websites mentioned above and then selected the miRNAs according to the seed match sequences. An entire network of circRNA/miRNA/gene interactions was delineated using Cytoscape 3.7.0 software (National Resource for Network Biology P41 GM103504, USA) (https://cytoscape.org/index.html) [33].

### Fluorescence in situ hybridization (FISH)

RNA FISH probes for the circGRAMD1B and miR-130a-3p sequences were designed and synthesized by GenePharma (Shanghai, China). Probe sequences are listed in Supplementary Table 2. In brief, Cy3 probes were specific to circGRAMD1B, and FAM probes were specific to miR-130a-3p. 4′,6-Diamidino-2-phenylindole (DAPI; Life Technologies, Carlsbad, CA, USA) was used to label cell nuclei. All the procedures were conducted according to the manufacturer’s instructions. All images were acquired on a confocal laser scanning microscope (LSM800, Carl Zeiss AG, Germany).

### Luciferase reporter assay

The wild-type (WT) or mutant-type (MUT) circGRAMD1B fragment and the 3′-untranslated region (UTR) of PTEN and p21 were subcloned downstream of the luciferase gene within the pmirGLO dual-luciferase reporter vector (Promega, Madison, WI, USA). HEK-293T cells grown in a 96-well plate were cotransfected with plasmids containing the 3′-UTR of the wild-type or mutant fragment from circGRAMD1B, PTEN, and p21 and its paired miR-130a-3p mimics using Lipofectamine 2000 (Invitrogen) according to the manufacturer’s protocol. After 48 h of transfection, firefly and Renilla luciferase activities were measured by using a dual-luciferase reporter assay system. The ratios of luminescence from firefly to Renilla luciferase were calculated, and each assay was repeated in 3 independent experiments.

### RNA immunoprecipitation (RIP)

RIP was conducted with a Magna RIP Kit (Millipore, Billerica, MA, USA) following the manufacturer’s protocols. In brief, AGS cells were harvested 48 h after transfection with miR-130a-3p mimics or the miR-NC and lysed in complete RNA lysis buffer. Then, cell lysates were incubated with magnetic beads conjugated to anti-Argonaute2 (AGO2; Millipore, Billerica, MA, USA) or a negative control IgG antibody at 4 °C for 4 h. Then, the beads were washed with the washing buffer. The immunoprecipitated RNA and protein were purified and enriched to detect the target RNAs and AGO2 by qRT-PCR and Western blot analyses.

### RNA pull-down assay

Biotin-labeled circGRAMD1B probes were designed and synthesized by GenePharma (Shanghai, China), and the probe sequences are listed in Supplementary Table 2. Approximately 1 × 10^7^ AGS cells were lysed with lysis buffer and incubated with specific circGRAMD1B probes for 2 h. The cell lysates were incubated with streptavidin-coated magnetic beads to pull down the biotin-labeled RNA complex for another 4 h. The beads were washed, and the RNA complex was extracted with TRIzol (Takara, Dalian, China). Then, the abundance of circGRAMD1B and miR-130a-3p was analyzed by qRT-PCR analysis.

### Western blot analysis

Total proteins from GC cells were extracted using RIPA buffer supplemented with protease and phosphatase inhibitors. Protein extractions were separated by 10% SDS-PAGE and transferred onto polyvinylidene fluoride (PVDF) membranes (Bio-Rad, CA, USA). The membranes were blocked with blocking solution and incubated with primary antibodies against PTEN (1:1000), p21 (1:1000), PCNA (1:1500), Ki-67 (1:1000), E-cadherin (1:1500), Vimentin (1:2000), β-actin or GAPDH (1:5000) (Abcam, Burlingame, CA, USA) at 4 °C overnight and then incubated with secondary antibodies (1:5000; Cell Signaling Technology, Beverly, MA, USA) at room temperature for 2 h. After washing, signals were detected using a chemiluminescence system (Bio-Rad, USA) and analyzed using Image Lab Software.

### Immunohistochemistry (IHC) and immunofluorescence (IF)

For the IHC assay, briefly, dissected tumors from human GC tissues or the mouse model experiment were paraffin-embedded and cut into 4 μm thick sections. After antigen retrieval, the sections were incubated with PTEN, p21, Ki-67, PCNA, E-cadherin, and Vimentin (Abcam) antibodies at 4 °C overnight. After incubation with the HRP-conjugated secondary antibody, the sections were stained with diaminoaniline (DAB) and then counterstained with Mayer’s haematoxylin. All images were acquired on a fluorescence microscope (Axio Imager A2, Carl Zeiss AG, Germany). For IF analysis, GC cells transfected with the circGRAMD1B-overexpressing plasmid or the silencing siRNA were planted onto climbing pieces, and some plates were cotransfected with miR-130a-3p mimics or inhibitors for 48 h. Cell climbing pieces were incubated with antibodies against PTEN and p21 (Abcam) overnight at 4 °C and with Cy3-labelled fluorescent secondary antibodies (Beyotime), dyed with DAPI and observed under a confocal laser scanning microscope (LSM800, Carl Zeiss AG, Germany).

### Animal experiments

Four-week-old BALB/c female nude mice were purchased from the National Laboratory Animal Center (Shanghai, China). The animal studies were performed in accordance with the institutional ethics guidelines for animal experiments, which were approved by the Animal Management Committee of Chongqing Medical University. For the in vivo tumor formation assay, approximately 1× 10^7^ MGC803 cells stably transfected with a lentivirus overexpressing circGRAMD1B and a control vector were injected subcutaneously into the axilla of the BALB/c nude mice (five mice per group). Tumor growth was monitored once a week and calculated as 0.5 × length × (width)^2^. After 4 weeks, the mice were euthanized, and the xenograft tumors were harvested for qRT- PCR and then fixed in 4% paraformaldehyde for IHC assay. For the tumor metastasis assay, the abovementioned cells were injected into nude mice via the tail vein (2 × 10^7^ MGC803 cells/ per mouse; four mice per group). After 5 weeks, the mice were euthanized, and the post-mortem examinations included the lung metastasis size or volume and number of nodules. The images of metastatic nodules in mouse lungs were captured and counted under a microscope, and then the metastatic nodules were used for qRT-PCR.

### Statistical analysis

All statistical analyses were conducted using SPSS 23.0 (SPSS, Chicago, IL, USA) or GraphPad Prism v7 (GraphPad Prism, Inc., La Jolla, CA, USA). Each experiment was performed at least in triplicate, and the data are presented as the mean ± SD of three independent experiments. Student’s t-test or ANOVA was used to compare the means of two or three groups. The correlation between circGRAMD1B expression and clinicopathological variables was calculated by the chi-square test or Fisher’s exact test. Pearson’s correlation coefficient analysis was used to analyze the correlations. P-value < 0.05 was taken as statistically significant.

## Supplementary Material

Supplementary Tables
