# Supplementary material for: Circular RNA circGRAMD1B inhibits gastric cancer progression by sponging miR-130a-3p and regulating PTEN and p21 expression
Source: Aging (Albany NY). 2019 Nov 13;11(21):9689–708. doi: 10.18632/aging.102414 (PMC6874462; doi:10.18632/aging.102414)
Supplement: Supplementary Tables [file aging-11-102414-s001.pdf]

SUPPLEMENTARY TABLES

Supplementary Table 1. Correlations between the proportion of circGRAMD1B and clinicopathological parameters in 60 GC patients.

Characteristics	No. of patients	circGRAMD1B		*P* value
		Low	High	
All cases	60	39	21	
Gender				
Male	44	30	14	0.392
Female	16	9	7	
Age				
≤65	39	26	13	0.712
>65	21	13	8	
Tumor size (cm)				
≤5	31	16	15	0.025*
>5	29	23	6	
Cancer location				
Upper	18	11	7	0.832
Middle	16	10	6	
Lower	26	18	8	
Differentiation grade				
Well-moderate	32	19	11	0.940
Poor-differentiation	28	18	10	
Carbohydrate antigen 19-9				
Negative	25	14	11	0.217
Positive	35	25	10	
T stage				
T1-T2	30	15	15	0.015*
T3-T4	30	24	6	
Lymph node status				
Negative	28	17	11	0.515
Positive	32	22	10	
Distant metastasis				
M0	48	29	19	0.185
M1	12	10	2	
TNM stage				
I-II	32	18	14	0.129
III-IV	28	21	7	

The TNM Staging System is based on the tumor (T), the extent of spread to the lymph nodes (N), and the presence of metastasis (M); *P < 0.05.

Supplementary Table 2. Primers, Probes, and siRNA information.

Primers/Probes	Sequence
GAPDH F	5′-CTTTGGTATCGTGGAAGGACTC-3′
GAPDH R	5′-GTAGAGGCAGGGATGATGTTCT-3′
circGRAMD1B F	5′- gcactgattcagaaaaggtgtta -3′
circGRAMD1B R	5′- cctgaaggagaatgtctctt-3′
GRAMD1B F	5′-CTACACAATCAATCGCTACACG-3′
GRAMD1B R	5′-CATCTCAGCCAAATAAGTGCTC-3′
PTEN F	5′-GACCAGAGACAAAAAGGGAGTA-3′
PTEN R	5′-ACAAACTGAGGATTGCAAGTTC-3′
P21 F	5′-GATGGAACTTCGACTTTGTCAC-3′
P21 R	5′-GTCCACATGGTCTTCCTCTG-3′
Ki-67 F	5′-CAGACATCAGGAGAGACTACAC-3′
Ki-67 R	5′-GTTAGACTTGCTGCTGAGTCTA-3′
PCNA F	5′-TAATTTCCTGTGCAAAAGACGG-3′
PCNA R	5′-AAGAAGTTCAGGTACCTCAGTG-3′
E-cadherin F	5′-AGTCACTGACACCAACGATAAT-3′
E-cadherin R	5′-ATCGTTGTTCACTGGATTTGTG-3′
Vimentin F	5′-GGACCAGCTAACCAACGACA-3′
Vimentin R	5′-AAGGTCAAGACGTGCCAGAG-3′
siRNA1-circGRAMD1B	5′-GAAAAGGTGTTAAGCCCCACC-3′
	5′-AGGCCACCTAACGCTTAAGAG-3′
siRNA2-circGRAMD1B	5′-AGAAAAGGTGTTAAGCCCCAC-3′
	5′-GAACCGACCAGCTTAAGATGA-3′
FISH-circGRAMD1B probe	5′-TGGGGCTTAACACCTTTTCTGAATCAGTGC-3′
FISH-miR-130a-3p probe	5′-ATGCCCTTTTAACATTGCACTG-3′
Pull down-circGRAMD1B probe	5′-TTGTAGGTGGGGCTTAACACCTTTTCTGAATCAGTGCAAA-3′
